# The Relation of Gynæcology to the Glands of Internal Secretion

**Published:** 1931

**Authors:** D. C. Rayner

**Affiliations:** Professor of Gynœcology and Obstetrics in the University of Bristol


### The Bristol Medico-Chirurgical Journal

*"Scire est nescire, nisi id me Scire alius sciret."*

SPRING, 1931.

### THE RELATION OF GYNÆCOLOGY TO THE GLANDS OF INTERNAL SECRETION.

The Presidential Address, delivered on 8th October, 1930, at the opening of the Fifty-eighth Session of the Bristol Medico-Chirurgical Society.

BY

D. C. Rayner, Ch.M., F.R.C.S.,

*Professor of Gynæcology and Obstetrics in the University of Bristol.*

My first and pleasant duty on occupying this Chair is to thank you for the very great honour which you have conferred upon me by electing me your President. I can assure you I greatly appreciate it, and will do my utmost to carry out the duties which the office entails. But I am met at the very outset with the difficulty of giving an inaugural address which shall be worthy to take its place beside some of

B

the able and eloquent addresses of past Presidents. However, I shall claim your indulgence for any shortcomings.

I propose to make a few remarks on "The Relation of Gynæcology to the Glands of Internal Secretion." There is an increasing mass of evidence that the female organism is peculiarly susceptible to physiological and pathological changes in the general endocrine system. Much of our knowledge, gleaned from the vast store of experimental and clinical observation in this new line of research, is contradictory and confusing. It will be necessary, therefore, to devote our attention chiefly to those facts which are most convincing and most generally accepted, with especial reference to those phases of the subject which relate particularly to the generative system.

Broadly speaking, each tissue of the body by means of its specific chemical sections is capable of affecting through the blood-stream other parts of the body ; but the glands with which we are principally concerned are those which produce hormones with especially powerful and important physiological functions. They comprise the ovary, the thyroid apparatus, the suprarenal system, and the pituitary body.

Although by the internal secretory theory the influence of distant organs on each other is based on a chemical reaction, instead of (as was formerly believed) on a direct nervous relationship, the nervous system does play an important part, for many of the phenomena produced by the internal secretions are brought about by chemical action on the sympathetic system of nerves. The intimate relation between the ductless glands and the sympathetic nerves is especially significant from a gynæcological standpoint, on account of the peculiarly sensitive character of the female nervous organization. This is particularly noticeable at the so-called critical periods of a woman's life, viz. at puberty, during menstruation, pregnancy and the climacteric. Although each gland produces its own particular hormone, it is important to remember that there is a certain amount of correlation between the various glands, with the result that if one of the glands is diseased, or injured or extirpated, the normal balance is upset, and the organism of the individual may be affected by the abnormal action of one or more distant glands of the group.

It was at one time thought that an internal secretion could only be elaborated by epithelial elements, but it has been discovered that structures of connective tissue origin are also capable of manufacturing an internal secretion. Examples of this are seen in the hypophysis, the suprarenals, and possibly the ovaries.

*The Ovary.*—That the ovary is a true organ of internal secretion is proved by substantial evidence, gained from observations made after removal of these organs, also from the transplantation of ovarian tissue and by the effects of injection of ovarian substances into the tissue.

Castration before sexual maturity causes a failure of genital development, and in adult life produces immediate regressive changes in the uterus, vagina, and external genitals, manifested by a well-marked atrophy of the parts. The exact nature of the ovarian secretion has not been determined, nor is it conclusively known in what part of the ovarian tissue the substance is manufactured. It seems probable that different portions of the ovary elaborate substances which have specific functions. There are two anatomical structures which may most reasonably be expected to be the source of hormones, viz. the corpus luteum and the follicle apparatus. It is probable that the follicle apparatus presides over the growth and nutrition of the genitals, and that it influences directly or indirectly the general bodily development of the individual.

In addition to the follicle apparatus, the corpus luteum has been shown to be the probable seat of manufacture of an important internal ovarian secretion. In its full development the corpus luteum presents the characteristic picture of an internal secretory gland, with large pale cells lying in close proximity to thin-walled blood-vessels, an appearance much like that of the adrenals. The corpus luteum does not develop until the age of puberty, and coincident with its appearance come the cyclical changes of menstruation and the possibility of fecundation, phenomena which disappear after the cessation of corpus luteum formation at the menopause.

It is probable that the interstitial cells of the ovary, corresponding morphologically with the cells of Leydig in the male, can also manufacture an internal secretion. It has been objected that the interstitial cells, being of connective tissue origin, are not of the true endosecretory type, which is usually epithelial in character. This objection is met by the fact that certain other connective tissue elements are known to possess endosecretory power, notably the suprarenal cortex and the cells of Leydig.

Clinical manifestations of disturbances of the glands of internal secretion are caused by deficient activity (hypofunction) or by abnormal increase of activity (hyperfunction) of the glandular secreting substance. In studying the effect of the removal or destruction of a given ductless gland, we must take into account not only the influence of the loss of its secretion on the organism, but also the changes wrought in the other members of the group, the balance of whose function has thus been disturbed.

*Hypofunction of the Ovary.*—Early castration prevents normal development of the genital system. It also produces changes in some of the other ductless glands, the most notable being the hypophysis, in which there takes place an increase in size of the anterior lobe. It is important to emphasize the fact that early castration does not transform the individual into the heterosexual type. The clinical signs associated with hypofunction of the ovary consist of late onset of menstruation with scanty flow, and often severe spasmodic dysmenorrhœa. It is probable that many of these changes are the result of disturbing the balance of the other ductless glands. From clinical observation it may be said that late castration in women has comparatively little effect, and that it does not entail the profound mental and physical changes in the organism of the individual that were formerly imputed to it. The amenorrhœa that is sometimes seen in fully-developed women, who have previously menstruated normally, is accounted for as a result of ovarian deficiency, a theory that finds strong confirmation in the almost immediate beneficial effect which ovarian extract sometimes has on these cases.

*Hyperfunction of the Ovary.*—Our present knowledge of the influence of hypersecretion of the ovary is only theoretical, and not sufficiently founded on scientific facts. Abnormal activity of the gland is supposed to be manifested by menorrhagias, and possibly by premature sexual development and over-fertility.

From a clinical standpoint the most important phase of the question is that which applies to those cases of uterine bleeding which cannot be satisfactorily accounted for on an anatomical basis, the so-called cases of uterine insufficiency in which there is severe menorrhagia without microscopic or macroscopic changes in the tissues of the uterus or adnexa. The belief that most so-called functional uterine bleeding is the result of hypersecretion of the ovary is based on the theory of the inter-relationship between ovarian secretion and menstruation. It is thought that in the ovaries is produced a substance that is passed over to and stored in the uterus and endometrium, which has the power of local dilatation of the blood-vessels, and of delaying or preventing the coagulation of the blood, thus bringing about increased or prolonged menstrual flow. The evidence, however, in support of this theory is not very convincing, and it is doubtful whether the ovarian hormone of the mature woman has any very important constitutional function beyond that of its specific action in the processes relating to reproduction.

*Ovarian Transplantation.*—Perhaps the most striking evidence that the ovary is an organ of internal secretion is seen in the effects produced by implantation and transplantation of ovarian tissue. It has been shown that in young castrated animals the genitalia may be made to develop normally at the time of maturity if ovarian tissue is implanted under the skin. By these experiments the ovarian influence by direct nerve connection is excluded, and it is clearly established that the ovary secretes a substance which acts chemically on distant organs through the agency of the blood-stream.

To the gynæcologist ovarian transplantation has been a subject of peculiar interest, in that it seemed to promise a solution of many pelvic problems. Practical results have, however, up to the present time been disappointing; but it is quite possible that with improved technique and with a greater knowledge of the ovarian functions some of the hopes for its usefulness may in time be realized.

In the treatment of sterility ovarian transplantation in the human female has been for the most part disappointing. Numerous cases of pregnancy following autoplastic grafting of ovarian tissue in the uterine cornua, after complete bilateral salpingectomy, have been reported, but in most of these cases the pregnancy has terminated in abortion.

*Ovarian Organotherapy.*—It is a matter of keen disappointment to clinicians that the new knowledge gained by science of the function and properties of the female sex organs has not led to a more effective therapy, but there are numerous reasons for this. In the first place the secretory power of the ovary in normal adult woman is directed chiefly to the pelvic organs for purposes of reproduction, and has little to do with other functions of her general organism. This is shown by the comparatively small harm that is done by the removal of the secretion. The ovary is not an organ of the same vital significance as the adrenals, the hypophysis and the thyroid, nor are its extracts as toxic as those of the other ductless glands. Moreover, it must be remembered that whereas in the body the ovarian secretion passes directly into the circulation, ovarian therapy when administered orally requires that the substance pass through the digestive apparatus, so that a chemical disturbance in the secretion, amounting possibly to destruction, is inevitable. More satisfactory results are, as a rule, obtained after hypodermic or intravenous injection. It is in the treatment of the vasomotor disturbances of the menopause that ovarian substance gives the best results.

The conclusions that may be drawn from ovarian therapy are: (1) Ovarian substance is a new specific in the treatment of hot flushes, and the vasomotor disturbances of the menopause ; (2) it is ineffective in permanent amenorrhœa with hypoplasia, but is useful in certain conditions of menstrual deficiency ; (3) it is occasionally useful in cases of essential dysmenorrhœa not associated with marked hypoplasia ; (4) in the treatment of sterility, instances of pregnancy following ovarian therapy are sufficiently common to suggest that in certain cases of deficient ovulation ovarian extract may effect a cure ; (4) preparations of the ovarian residue are more efficacious than those of the corpus luteum alone, seeing they contain the more potent hormone from the follicular apparatus, and are free from the toxic and inhibitory element that characterizes the corpus luteum ; (4) there is sufficient chemical and experimental evidence to encourage the hope that ovarian extracts, now admittedly feeble and inconstant in their action, will, under the direction of research, be produced of such potency as to be of constant specific value in the treatment of certain functional disorders.

*The Hypophysis.*—There has been demonstrated an intimate functional relationship between the hypophysis and the sex glands, hence the subject is of special interest to the gynæcologist. Clinically changes in the anterior lobe produce characteristic reactions in the general organism, as is exemplified by such diseases as gigantism and acromegaly. Extracts of the anterior lobe exercise a definite influence on the sexual glands.

Extracts of the posterior lobe have a specific action on certain unstriped muscular systems, more particularly the uterus. The reciprocal action between the hypophysis and the sex glands is manifested in numerous ways. Very striking is the influence of pregnancy on the size and weight of the hypophysis.

Erdheim and Stummer made a systematic study of the hypophysis of women who had died during pregnancy, and found that the gland always becomes hypertrophied during the pregnant state. They showed that the hypertrophy takes place only in the anterior lobe, and that the posterior lobe not only does not share in the overgrowth but actually suffers a certain amount of compression.

The influence exerted on the sexual glands by changes in the hypophysis is manifested through the effect of increased or diminished function of the gland. *Hyperfunction* is due to increased activity of the anterior lobe, consequent upon adenomatous enlargement, or hyperplasia of that portion of the gland. If under the influence of a diseased condition hyperplasia of the anterior lobe occurs before puberty the ultimate result is gigantism. If the hyperplasia takes place after maturity acromegaly ensues. If *hypofunction* or diminished secretion occurs before puberty it causes retardation in skeletal growth, and the reproductive organs and secondary sex characters are infantile. When the condition develops after maturity similar though less marked changes appear, viz. adiposity, retrogressive changes in the sex organs, with irregularities of function and various abnormalities of metabolism.

*Organotherapy.* — Pituitrin, the extract of the posterior lobe, has proved of the greatest value, particularly in obstetrics. Although the extract has a stimulating influence on unstriped muscle generally, it seems to exert a specific action on the parturient uterus. Its use, however, requires considerable care and judgment, as its indiscriminate employment may involve danger to both mother and child.

Pituitrin is also a useful adjunct in the treatment of intestinal paresis and distension following abdominal operation.

The experimental work that showed the influence of the anterior lobe of the pituitary on growth and sexual development raised great hopes that a potent agent had been discovered for the treatment of delayed development, infantilism, hyposexuality and amenorrhœa, but these hopes have in large measure been doomed to disappointment. Extracts of the anterior lobe have proved to be feeble in effect.

*The Thyroid.*—In the relationship of the ductless glands to the general organism, the thyroid from the standpoint of the clinician has assumed a position of the highest importance. In the case of the ovary and the hypophysis we have been dealing with organs whose active constituents are so subtle that our knowledge of them is vague and confused. The thyroid, on the contrary, possesses a hormone which not only manifests itself in unmistakable ways, but is capable of being replaced by organ extracts. The function of the gland is far-reaching throughout the entire organism, and represents a single chemical reaction, which, as Kendall says, is necessary for the life of every cell and tissue of the body. It powerfully influences cell oxidation, so that when the gland is over-active it "gradually consumes the tissues as would glowing heat," an effect familiarly seen in Graves's disease. When the function of the thyroid is deficient it is manifested by various vital forms of physical and mental sluggishness, the maximum expression of which appears in myxœdema and cretinism.

The variations in size of the thyroid gland during certain phases of the female sexual life have been observed since the earliest times. At puberty the thyroid at times takes on a marked enlargement, and it is probable that many of the puberty symptoms in girls, such as the tendency to palpitation and the evidences of vasomotor disturbances, may be due to a hypersecretion of the gland at this period.

Experiments have shown a certain antagonism between the ovary and the thyroid gland. Animals that have been castrated early exhibit an abnormal length of certain bones, especially the tibia, whereas in animals from which the thyroid has been extirpated these same bones are exceedingly short.

The early extirpation of the thyroid produces a certain amount of degenerative change in the ovaries, delays the time of puberty and greatly limits the productivity of the individual. Women with diseased thyroids usually have menstrual disorders. Kocher has observed that patients on whom a too radical goitre operation has been performed suffer from menorrhagia, and he has treated such cases successfully with thyroid extract.

That the relationship between the thyroid and the genitals is not fully understood is shown by the fact that in some cases of myxœdema there is amenorrhœa, and that thyroid extract works beneficially for the conditions both of menorrhagia and of amenorrhœa when the gland is diseased. The swelling of the thyroid is most noticeable during pregnancy, and it appears to be due to hypertrophy rather than to hyperæmia of the organ, because of its amenability to iodine treatment. Lange has made the interesting observation that women in whom the gland does not swell tend to exhibit a renal albuminuria, and expressed the belief that swelling of the thyroid acts as a protection against certain poisons which are set free as a result of pregnancy, and which without the protection of the secretion of the hypertrophied thyroid are injurious to the kidneys.

The influence of the climacteric on the thyroid gland is little known, and the subject is largely a matter of speculation; it may be that the neuropathic and vasomotor symptoms of the change of life are the result of hyperactivity of the thyroid gland.

To cases in which the sexual system is definitely influenced by the thyroid Berchardt has given the name of *thyrosexual insufficiency*. This term is applied to the malady when it occurs at the so-called sexual crises, *i.e.* puberty, the puerperium and the climacteric. At puberty in girls the condition is associated with infantilism, from which they do not always recover. Puerperal thyrosexual insufficiency is signalized by amenorrhœa, access of fat, genital atrophy, and sometimes atrophy of the breasts. The climacteric type of thyrosexual insufficiency may represent a correlated gland change. The marked bodily and facial changes that often take place about the time of the menopause, which are popularly supposed to be the result of the loss of ovarian activity, represent more likely the ageing effects of diminished thyroid function.

*The Adrenals.*—The two systems of the suprarenal apparatus have each an independent internal secretory function. Most of our present knowledge of the specific secretion of the two parts of the suprarenals relates to the medullary system which elaborates the hormone called adrenalin. Of especial interest is the fact that the hormone of the suprarenal system is greatly influenced by conditions of emotional disturbance. In view of the emotional liability that characterizes woman's nature, especially at such critical periods as puberty, menstruation, pregnancy and the menopause, and the organic disturbances that occur at such times, there undoubtedly exists through the medium of the emotions an important relationship between the organs of generation and the internal secretion of the adrenals. It has been observed that animals endowed with the strongest sexual powers are also possessed of markedly developed suprarenals. Negroes are distinguished by a greater development of both sexual and suprarenal glands than is seen in white races. There is considerable evidence that the adrenal tissue of both cortex and medulla is hypertrophied during pregnancy, and probably at menstruation.

I think the facts I have enumerated show the very profound influence exerted on the pelvic organs by the endocrine glands, and in this connection I should like to acknowledge my indebtedness to Professor Graves's excellent work on gynæcology. At the present time we are beginning to realize more and more that many of the so-called neurotic and hysterical symptoms met with in gynæcological patients are really associated with some organic lesion, and it seems not unlikely that the clue to many of these baffling symptoms will be found in some pathological change in the endocrine system.

